# The multiplex bead array approach to identifying serum biomarkers associated with breast cancer

**DOI:** 10.1186/bcr2247

**Published:** 2009-04-28

**Authors:** Byoung Kwon Kim, Jong Won Lee, Pil Je Park, Yong Sung Shin, Won Young Lee, Kyung Ae Lee, Sena Ye, Heesun Hyun, Kyung Nam Kang, Donghwa Yeo, Youngdai Kim, Sung Yup Ohn, Dong Young Noh, Chul Woo Kim

**Affiliations:** 1 Department of Laboratory Medicine and Pathology, The Armed Forces Capital Hospital, 2nd street, Yul-dong, Bundnag-gu, Sungnam city, Gyeonggi-do, 434-040, Korea; 2 Department of Surgery, Seoul National University College of Medicine, Daehak street, 28 Yeongeon-dong, Jongno-gu, Seoul 110-799, Korea; 3 BioInfra Inc., Cancer Research Institute, Seoul National University College of Medicine, Daehak street, 28 Yeongeon-dong, Jongno-gu, Seoul 110-799, Korea; 4 Department of Statistics, Seoul National University, Gwanak street, Gwanak-gu, Seoul 151-742, Korea; 5 Department of Computer Engineering, College of Engineering, Korea Aerospace University, 100 Hanggondae street, Hwajeon-dong, Deogyangu, Goyang city, Gyeonggi-do, 412-791, Korea; 6 Department of Pathology, Cancer Research Institute, Tumor Immunity Medical Research Center, Seoul National University College of Medicine, Daehak street, 28 Yeongeon-dong, Jongno-gu, Seoul 110-799, Korea

## Abstract

**Introduction:**

Breast cancer is the most common type of cancer seen in women in western countries. Thus, diagnostic modalities sensitive to early-stage breast cancer are needed. Antibody-based array platforms of a data-driven type, which are expected to facilitate more rapid and sensitive detection of novel biomarkers, have emerged as a direct, rapid means for profiling cancer-specific signatures using small samples. In line with this concept, our group constructed an antibody bead array panel for 35 analytes that were selected during the discovery step. This study was aimed at testing the performance of this 35-plex array panel in profiling signatures specific for primary non-metastatic breast cancer and validating its diagnostic utility in this independent population.

**Methods:**

Thirty-five analytes were selected from more than 50 markers through screening steps using a serum bank consisting of 4,500 samples from various types of cancer. An antibody-bead array of 35 markers was constructed using the Luminex™ bead array platform. A study population consisting of 98 breast cancer patients and 96 normal subjects was analysed using this panel. Multivariate classification algorithms were used to find discriminating biomarkers and validated with another independent population of 90 breast cancer and 79 healthy controls.

**Results:**

Serum concentrations of epidermal growth factor, soluble CD40-ligand and proapolipoprotein A1 were increased in breast cancer patients. High-molecular-weight-kininogen, apolipoprotein A1, soluble vascular cell adhesion molecule-1, plasminogen activator inhibitor-1, vitamin-D binding protein and vitronectin were decreased in the cancer group. Multivariate classification algorithms distinguished breast cancer patients from the normal population with high accuracy (91.8% with random forest, 91.5% with support vector machine, 87.6% with linear discriminant analysis). Combinatorial markers also detected breast cancer at an early stage with greater sensitivity.

**Conclusions:**

The current study demonstrated the usefulness of the antibody-bead array approach in finding signatures specific for primary non-metastatic breast cancer and illustrated the potential for early, high sensitivity detection of breast cancer. Further validation is required before array-based technology is used routinely for early detection of breast cancer.

## Introduction

Breast cancer is the most common malignant disease in women in western countries, comprising approximately 35% of all cancers [1]. The incidence of breast cancer has increased over the past few decades, probably due to earlier diagnosis, and mortality has been gradually reducing [2]. Nonetheless, prevention and early detection of breast cancer are two major issues of consideration for cancer epidemiologists and clinicians because radical treatment can greatly reduce breast cancer-related mortality if breast cancer is detected at an early stage [3]. Despite the use of mammography as a routine screening method for women 40 years of age and older, the effectiveness of this procedure in reducing overall population mortality is still being investigated [4]. Other diagnostic modalities that can improve diagnostic power in combination with conventional methods are required for strategic management of the disease and improvement of the overall mortality rate.

Biomarker research in easy-to-access biological fluids from cancer patients is expected to open up a new era in the field of cancer research and cancer diagnostics. Extensive searches have revealed several breast cancer-specific markers: MUC-1 family mucin glucoproteins like CA 15.3, BR27.29 (or CA27.29), and mucin-like carcinoma-associated antigen, CA 549, carcinoembryonic antigen (CEA), serum human epidermal growth factor receptor (HER) 2/c-erbB-2, cytokines and cytokeratin fragments [5-10]. Although these markers are not used for the purposes of screening and early diagnosis, they play a complementary role in staging work-up at initial presentation as indicated in the guidelines issued by the European Group on Tumor Markers (EGTM) [11] and the Food and Drug Administration [12].

Recent advancements in high-throughput platforms and information technology have ushered in the data-driven approach, which has emerged as a powerful and efficient way of conducting biomarker research and finding novel biomarkers. In the field of proteomics, the classical approach uses two-dimensional polyacrylamide gel electrophoresis (2D-PAGE) for comparing multiple protein profiles. However, this method has problems such as poor reproducibility and low throughput. Recent advances in mass spectrometry (MS), such as matrix-assisted laser desorption/ionisation (MALDI) time-of-flight MS, offer an alternative to 2D-PAGE [13]. However, some limitations in MALDI, such as extensive sample preparation and high signal background problems resulting from inorganic and organic contaminants, have hindered its wider use as a high-throughput screening tool to find useful proteins in complex biological samples. The development of surface-enhanced laser desorption/ionisation time-of-flight (SELDI-TOF) MS has largely overcome these limitations [14]. In the field of breast cancer research, Li and colleagues performed a pioneering study using SELDI-TOF and found potential biomarkers for detection of breast cancer, designating the peaks as BC1 (4.3 kDa), BC2 (8.1 kDa) and BC3 (8.9 kDa) [15]. Later, some of these were identified as fragments of serum complement protein, but these results are awaiting further validation.

Antibody-based microarray is also one of the data-driven approaches in proteomics that is likely to play an increasing role in the discovery of disease-specific signatures [16]. The spectrum of chemical biomarker information that can be elucidated using this method is relatively limited compared with that obtained using MS. However, the antibody-array platform bypasses the identification step for individual markers, making this a faster and more direct method for profiling protein expression and translating this information [17,18]. Furthermore, a combinatory strategy for utilising markers and statistics has been suggested to increase predictive power in cancer diagnosis, which re-energises the search for novel cancer-related biomarker signatures [19,20].

In line with this concept, Carlsson and colleagues adopted a planar array platform using single-chain variable fragment (scFv) targeting for more than 60 target antigens and found a serum protein signature that distinguishes breast cancer patients from normal subjects with high diagnostic accuracy [21]. Recently, the bead-array platform was also successfully applied to identify serum profiles predicting responses to neoadjuvant chemotherapy in locally advanced breast cancer [22].

Recently, our group constructed an antibody-based bead array panel consisting of 35 serum proteins via an extensive screening process using 4500 serum samples from various cancer patients. We report the characteristic serum profiles associated with breast cancer as revealed by application of this panel in an independent group of patients with mostly primary, non-metastatic disease and validate the diagnostic performance of these combinatorial markers.

## Materials and methods

### Study samples

Two sets of population were constructed. A training set (set 1) for selecting prediction biomarkers consisted of 194 people (98 breast cancer patients and 96 normal controls). The other independent set (set 2), consisting of 169 people (90 breast cancer patients and 79 normal controls), was used for validation of selected predictors from the initial set. In each set, cancer and control populations were age-matched. Serum samples of breast cancer patients were obtained before any type of surgical procedures. None of the patients had a family history of breast cancer. Serum samples for the controls were obtained from normal female subjects who voluntarily enrolled in the cancer screening program of Seoul National University Hospital and had no abnormalities identified on physical examination, routine blood testing or mammography. A complete medical history was obtained for each patient, including medication, menstrual history, menopause, alcohol consumption and smoking. All blood samples were collected before any type of surgical or medical intervention was performed. Peripheral blood was collected using 5 ml syringes and stored in SST™ II tubes (Becton Dickinson, Franklin Lakes, NJ, USA) at room temperature for one hour. Samples were centrifuged at 3000 g for five minutes, and the supernatants were collected and stored at -80°C before the assay was performed. Samples were drawn after obtaining informed consent from all patients. The study protocol was reviewed and approved by Institutional Review Board at Seoul National University Hospital (approval No. C-0512-502-163).

### Construction of 35-plex bead array panel

**Figure 1:**
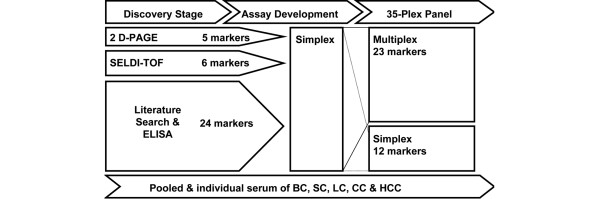
Procedure for constructing the 35-plex panel. Through using 4500 serum samples from cancer patients, five markers were discovered and identified through two-dimensional polyacrylamide gel electrophoresis (2D-PAGE), six markers through surface-enhanced laser desorption/ionisation time-of-flight (SELDI-TOF) and 24 markers through conventional sandwich ELISA method. After optimisation in capturing and detecting the antibody pair for a target analyte, the capturing antibody was conjugated with beads, and the simplex kit was validated for its dynamic range, recovery rate, parallelism with standard curve, interference, matrix effect, and lower and upper limits of detection. Twenty-three simplex kits could be grouped together according to dilution factor and absence of cross reactivity. The remaining 12 were left and used as a simplex kit. BC = breast cancer; SC = stomach cancer; LC = lung cancer; CC = colon cancer; HCC = hepatocellular carcinoma.

The antigen panel consisted of 35 analytes (Table 1). Thirty-five markers were chosen from 51 original markers (Figure 1). A serum bank had been constructed for determination of cancer biomarkers. The serum bank contained approximately 4500 samples from five types of cancer: breast, colon, stomach, liver and lung. Alpha-1-antitrypin, pro-apolipoprotein (proApo) A1, apolipoprotein (Apo) A4, haptoglobin α, and transthyretin were discovered using 2D-PAGE. ApoH, β2-microglobulin, vitamin D-binding protein, C-reactive protein (CRP), free haemoglobin and serum amyloid A were discovered using SELDI-TOF MS. These 11 markers were regarded as candidate markers with 40 markers selected through a literature search. The list of 51 candidate markers at this stage was as follows: adiponectin, alpha-2 macroglobulin (A2M), alpha-fetoprotein (AFP), alpha-1 antitrypsin (A1AT), ApoA1, proApoA1, ApoA2, ApoA4, ApoC2, ApoC3, ApoH, ApoJ (clusterin), beta-2 microglobulin, ferritin, CA-125, CA19-9, CA72-4, CEA, cathepsin B, chromogranin A, CRP, D-dimer, epidermal growth factor (EGF), galectin 3, gelsolin, haptoglobin alpha, total haptoglobin, free haemoglobin, heat shock protein (HSP) 27, HSP 70, high-molecular-weight kininogen (HMWK), human cervical cancer oncogene-1, IL-1 beta, IL-1 receptor alpha, IL-6, IL-8, monocyte chemoattractant protein-1, macrophage inflammatory protein-1, myeloperoxidase (MPO), neuron specific enolase, retinol binding protein 4, total plasminogen activator inhibitor (PAI)-1, total prostate specific antigen (PSA), free PSA, pro-gastrin releasing peptide, soluble CD-40 ligand (sCD40L), soluble intercellular adhesion molecule (sICAM)-1, soluble vascular cell adhesion molecule (sVCAM)-1, serum amyloid A (SAA), transthyretin, vitamin-D binding protein (VDBP) and vitronectin. The sandwich ELISA method for individual analytes was used as a validation method, again using samples from the serum bank. Markers with significantly different mean serum concentrations in cancer patients and normal subjects were selected and subjected to the 35-plex bead array panel.

**Table 1:** List of biomarkers in 35-plex panel

**Marker list**	
**Oncofetal protein**	**Acute phase protein**
Alpha-fetoprotein (AFP)	Alpha-1-antitrypin (A1AT)^a^
Carcinoembryonic antigen (CEA)	Alpha-2 macroglobulin (A2M)^c^
Cancer antigen 125 (CA125)^c^	C-reactive protein (CRP)^b^
Cancer antigen 19-9 (CA19-9)^c^	D-dimer (DD)^c^
Prostate-specific antigen (PSA)^c^	Haptoglobin α (Hp)^a^
**Cytokine**	Serum amyloid A (SAA)^b^
Interleukin-1β (IL-1β)^c^	Transthyretin (TTR)^a^
Interleukin-1 receptor α (IL-Rα)^c^	**Coagulation/thrombosis**
Interleukin-6 (IL-6)^c^	Haemoglobin (Hg)^b^
Interleukin-8 (IL-8)^c^	High-molecular-weight kininogen (HMWK)^c^
Monocyte chemotactic protein-1 (MCP-1)^c^	Plasminogen activator inhibitor-1 (PAI-1)^c^
Monocyte inflammatory protein-1α (MIP-1α)^c^	**Metabolism**
**Immune/inflammation**	Apolipoprotein A1 (ApoA1)^c^
Soluble CD40 ligand (sCD40L)^c^	Pro-apolipoprotein A1 (proApoA1)^a^
β2-microglobulin (β 2M)^b^	Apolipoprotein A4 (ApoA4)^a^
Myeloperoxidase (MPO)^c^	Apolipoprotein H (ApoH)^b^
**Factor/hormone**	**Carrier**
Epidermal growth factor (EGF)^c^	Vitamin D-binding protein (VDBP)^b^
Adiponectin^c^	**Enzyme**
**Adhesion**	Cathepsin B (CB)^c^
Vitronectin (VN)^c^	
Soluble vascular cell adhesion molecule-1 (sVCAM-1)^c^	
Soluble intercellular cell adhesion molecule-1 (sICAM-1)^c^	

^a^Markers were discovered through two-dimensional polyacrylamide gel electrophoresis (2D-PAGE) using the serum of breast cancer patients. ^b^Markers were discovered through surface-enhanced laser desorption/ionisation time-of-flight (SELDI-TOF) mass spectrometry. ^c^Markers were selected using literature search.

Bead array kits or antibodies for the construction of the 35-plex panel were purchased from the following manufacturers: Abcam (Cambridge, UK), Bethyl (Montgomery, TX, USA), Biodesign International (Saco, ME, USA), BoditechMed (Chuncheon, Korea), Chemicon (Temecula, CA, USA), Dako (Glostrup, Denmark), EMD Chemicals Inc. (San Diego, CA, USA), Fitzgerald (Concord, MA, USA), HyTest (Turku, Finland), Linco Research, Inc. (St. Charles, MO, USA), Rules-based Medicine (Austin, TX, USA), R&D Systems (Minneapolis, MN, USA), Santa Cruz Biotechnology Inc. (Santa Cruz, CA, USA), Sigma-Aldrich (St Louis, MO, USA) and US Biological (Swampscott, MA, USA).

### Multiplex assay procedure

Multiplex assay was performed using the following procedure: a 96-well filter-plate (Millipore, Billerica, MA, USA) was blocked with PBS (pH 7.4) with 2% BSA. Twenty microliters of standard curve sample, prediluted control samples and patient samples were dispensed into the wells in duplicate. Twenty microliters of primary antibody-bead mixture were added into each well and incubated at room temperature for one hour. Twenty microliters of detection antibodies with biotinylation and 20 μL of streptavidin-phycoerythrin were added and incubated at room temperature for one hour. Each step was followed by a double washing step using 0.05% Tween-20 in PBS (PBST) with vacuum manifold (Millipore Corp., Billerica, MA, USA).

After the final washing step, samples were resuspended with 100 μL of PBST and read using a Luminex-200™ (Luminex Inc., Austin, TX, USA). The standard curve was calculated using five-parametric-curve fitting, and results were analysed using Beadview software (Upstate Biotechnology Inc., Lake Placid, NY, USA). Markers were grouped together according to dilution factor after cross-reactivity was checked across all analytes. Control samples at two levels in the dynamic range of the standard curve were run together in duplicate for quality control throughout the study. Intra-assay precision ranged from 2 to 16%, and inter-assay precision ranged from 6 to 19% during the experiment. The acceptance criteria for each individual run followed Westgard's rule [23].

### Bioinformatics and statistics

The values of markers were transformed into log values before analysis using a multivariate classification algorithm. As an initial step, principal component analysis (PCA) was performed using information related to the concentration of all 35 markers, in order to study clustering of breast cancer and normal subjects. Random forest (RF), support vector machine (SVM) and linear discriminant analysis (LDA) were the multivariate algorithms used. Among the 196 cases, two-thirds of the cases from the breast cancer and normal groups were randomly assigned to training sets, and the remaining one-third were assigned to test sets. We compared the prediction performances obtained from 50 randomly partitioned data sets. Classification models with selected predictors obtained from the experiment with set 1 were validated again with set 2. A receiver operating characteristic (ROC) curve was constructed, and the area under the curve (AUC) was calculated using each algorithm. We extracted a classifier consisting of a subset of protein markers yielding the best classification performance in the test sets. A student's t-test (two-sided) was performed to compare the mean serum marker levels among groups stratified by clinical and pathological variables, and Pearson's correlation was performed to compare maximum tumour length and number of lymph node metastases with serum biomarker levels. All calculations were performed using the R program package (Wirtschafts universität, Wien, Austria) [24].

## Results

### Analysis of differentially expressed serum makers in patients with breast cancer and in normal subjects

The mean serum concentrations for individual analytes were compared between patients with breast cancer and those without breast cancer. Among the 35 analytes, EGF, sCD40L and proApoA1 showed higher serum concentrations in breast cancer patients than in normal subjects (Table 2). HMWK, ApoA1, PAI-1, VDBP and vitronectin levels were significantly decreased in cancer patients. EGF showed the highest AUC value (0.89) and exhibited a diagnostic accuracy of 82.3%, sensitivity of 94.0% and specificity of 70.6% as a single marker.

**Table 2:** Summary of differentially expressed serum markers between breast cancer and control subjects

Marker	Breast cancer (Mean ± SD)	Normal (Mean ± SD)	*P *value^a^	AUC
EGF (pg/ml)	325.14 ± 208.39	76.67 ± 71.42		
sCD40L (pg/ml)	11226.31 ± 71363.00	32.17 ± 30.35	< 0.001	0.85
HMWK (ug/ml)	19.65 ± 13.49	33.98 ± 15.15	< 0.001	0.76
ApoA1 (ng/ml)	224.36 ± 94.02	322.70 ± 136.80	< 0.001	0.72
sVCAM-1 (ng/ml)	728.05 ± 170.55	832.57 ± 162.23	< 0.001	0.69
PAI-1 (ng/ml)	18.97 ± 6.51	23.38 ± 9.25	< 0.001	0.65
ProApoA1 (ng/ml)	26.74 ± 9823.61	22.14 ± 8.21	< 0.001	0.65
VDBP (ng/ml)	173.65 ± 36.15	191.63 ± 43.57	< 0.001	0.65
VN (ng/ml)	4848.98 ± 2626.57	5546.35 ± 2168.00	0.001	0.64
D-dimer (ng/ml)	1249.58 ± 259.30	539.70 ± 417.99	0.0340	0.59
A1AT (ng/ml)	3594.23 ± 5875.33	3143.05 ± 16616.57	0.808	0.51
CRP (ng/ml)	5154.88 ± 39551.30	2860.52 ± 16501.80	0.818	0.51
AFP (ng/ml)	0.81 ± 0.49	0.76 ± 0.53	0.376	0.54
CEA (ng/ml)	12.36 ± 8.75	10.24 ± 5.11	0.144	0.56
PSA (ng/ml)	0.02 ± 0.05	0.02 ± 0.1	0.750	0.51
CA125 (U/ml)	16.76 ± 71.52	4.91 ± 5.71	0.465	0.53
CA19-9 (U/ml)	60.17 ± 151.07	25.02 ± 20.67	0.016	0.60
B2M (ng/ml)	692.36 ± 166.13	720.68 ± 162.14	0.125	0.56
A2M (ng/ml)	74.00 ± 28.05	85.03 ± 34.28	0.010	0.61
Adiponectin (ng/ml)	12802.04 ± 10393.99	14358.12 ± 6563.28	0.014	0.60
MPO (pg/ml)	146459.9 ± 142930.01	112079.27 ± 100310.31	0.233	0.55
sICAM-1 (ng/ml)	162.31 ± 43.77	173.87 ± 57.84	0.171	0.56
IL-1β (pg/ml)	4.06 ± 11.12	2.74 ± 4.2	0.790	0.51
IL-1Rα (pg/ml)	269.47 ± 361.27	155.37 ± 207.52	0.008	0.61
IL-6 (pg/ml)	36.66 ± 94.93	33.27 ± 75.37	0.720	0.51
IL-8 (pg/ml)	450.89 ± 2970.97	24.24 ± 45.59	0.424	0.53
MCP-1 (pg/ml)	273.38 ± 312.79	280.57 ± 103.93	0.015	0.60
MIP-1α (pg/ml)	76.44 ± 225.17	56.39 ± 88.25	0.224	0.55
ApoA4 (ng/ml)	10382.14 ± 3924.34	10742.81 ± 3755.76	0.544	0.53
TTR (ng/ml)	177.40 ± 58.38	198.27 ± 65.85	0.029	0.59
Hp (ng/ml)	1080.38 ± 662.48	921.74 ± 448.23	0.197	0.55
SAA (ng/ml)	4261.8 ± 9028.56	2872.43 ± 5133.39	0.155	0.56
Hg (ng/ml)	249.95 ± 100.58	82 ± 44.23	< 0.001	0.66
CB (ng/ml)	943.79 ± 110.01	1081.19 ± 847.39	0.003	0.62
ApoH (ng/ml)	125.67 ± 131.69	128.54 ± 31.32	0.520	0.53

^a^Specific false discovery rate at a 1% level.

A1AT = alpha-1 antitrypsin; A2M = alpha-2 macroglobulin; AFP = alpha-fetoprotein; Apo = apolipoprotein; AUC = area under the curve; B2M = beta-2 macroglobulin; CA = cancer antigen; CB = cathepsin B; CEA = carcinoembryonic antigen; CRP = C-reactive protein; EGF = epidermal growth factor; Hg = haemoglobin; HMWK = high-molecular-weight kininogen; Hp = haptoglobin; IL = interleukin; MCP = monocyte chemotactic protein; MIP = monocyte inflammatory protein; MPO = myeloperoxidase; PAI = plasminogen activator inhibitor; ProApo = proapolipoprotein; PSA = prostate specific antigen; SAA = serum amyloid A; sCD40L = soluble CD-40 ligand; SD = standard deviation; ICAM = intercellular adhesion molecule; sVCAM = soluble vascular cell adhesion molecule; TTR = transthyretin;VDBP = vitamin-D binding protein; VN = vitronectin.

### Multivariate classification using combinatorial biomarkers specific for breast cancer

**Figure 2:**
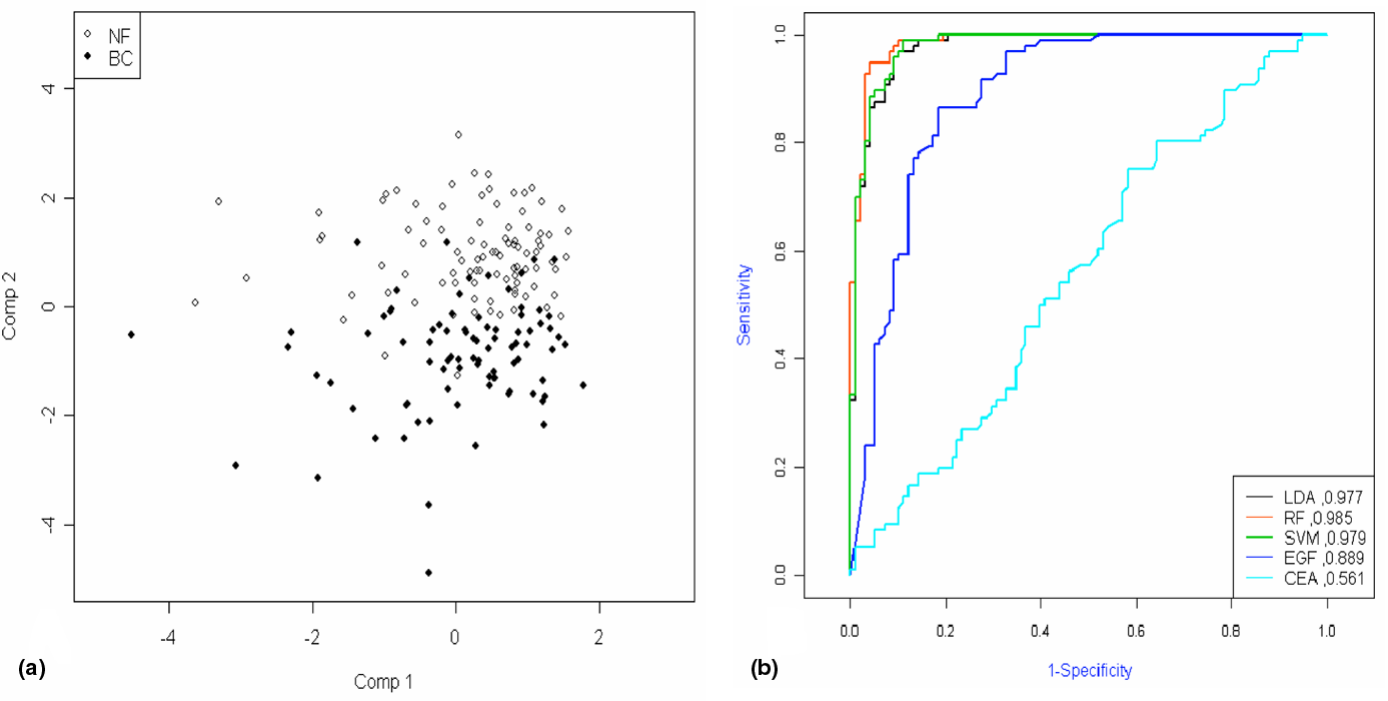
Classification performance of combinatorial markers identified through 35-plex panel assay. **(a) **Principal component analysis (PCA) with 35 markers showed clustering and separation of breast cancer patients (closed circle) and normal subjects (open circle) in the PCA chart using principal component (Comp) 1 and 2. NF = normal female; BC = breast cancer. **(b) **The area under the curve was calculated for combinatorial markers and a single marker, and compared using a receiver operating curve. CEA = carcinoembryonic antigen; EGF = epidermal growth factor; LDA = linear discriminant analysis; RF = random forests; SVM = support vector machine.

In order to geometrically interpret and determine if breast cancer patients could be segregated from normal subjects, PCA analysis was performed using all the data related to serum levels of the 35 markers. Principal components deduced from variance-covariance structures of these markers separated these two groups using the top two principal components (Figure 2a). In order to find classifiers that distinguish breast cancer patients from healthy people, multivariate classification analysis was performed using RF, SVM and LDA. In the interest of constructing models and selecting predictors, two-thirds of the original set was assigned to training sets. After training, each model consisting of different sets of classifiers was validated through the test set. The accuracy and classification error for each model were calculated in each training and test set. The calculated averages are summarised in Table 3. RF, SVM and LDA classified breast cancer and normal subjects with a mean accuracy of 91.8%, 91.5% and 87.6%, respectively. For the validation of this model, an independent validation set consisting of 169 persons was analysed using the same model and predictors. The calculated averages were similar to those obtained from the original set (Table 3). The combination of markers showing the highest diagnostic accuracy was very similar in all three models. EGF, sCD40L, HMWK, ApoA1, PAI-1 and VDBP were consistently selected by all three algorithms. D-dimer and vitronectin were chosen by RF and SVM. Pre-treatment serum levels of CA15-3 were available in 96 patients, and serum levels of tissue polypeptide antigen were available in 77 patients. When the sensitivity of combinatorial markers was compared with that of single markers, multi-classifiers showed improvement not only in overall sensitivity for total patients but also in sensitivity for early-stage disease (Table 4).

**Table 3:** Diagnostic performance of three classification algorithms using combinatorial markers

Algorithm	Marker combination	Accuracy (%)		Sensitivity (%)		Specificity (%)	
		
		Train	Validation	Train	Validation	Train	Validation
RF	EGF, sCD40L, HMWK, ApoA1, PAI-1, DD, VDBP, VN	91.8	93.8	89.8	92.8	93.7	94.7
SVM	EGF, sCD40L, HMWK, ApoA1, PAI-1, DD, VDBP, VN	91.5	88.4	89.5	87.5	93.4	89.3
LDA	EGF, sCD40L, HMWK, ApoA1, PAI-1, VDBP	87.6	87.4	84.8	88.4	90.4	86.1

Apo = apolipoprotein; DD = D-dimer; EGF = epidermal growth factor; HMWK = high-molecular-weight kininogen; LDA = linear discriminant analysis; PAI-1 = plasminogen activator inhibitor-1; RF = random forests; sCD40L = soluble CD-40 ligand; SVM = support vector machine; VDBP = vitamin-D binding protein.

**Table 4:** Comparison of sensitivity of combinatorial markers vs. single marker

		Stage I to II	Stage III to IV	Total cases
		
Classification method		Sensitivity (%)	Sensitivity (%)	Sensitivity (%)
Algorithm	RF	86.3(44/51)	93.6(44/47)	89.8
	SVM	90.2(46/51)	93.6(44/47)	91.8
	LDA	82.4(42/51)	85.1(40/47)	83.7
Single marker	CA15-3*	0 (0/49)	6.4 (3/47)	3.1 (3/96)
	TPA*	23.1 (9/39)	29.0 (11/38)	26.0 (20/77)

*Cut-off levels of CA15-3 and TPA were 27 U/ml and 95 U/L, respectively.

CA = cancer antigen; LDA = linear discriminant analysis; RF = random forests; SVM = support vector machine; TPA = tissue polypeptide antigen.

### Comparison of biomarkers with clinico-pathological parameters of breast cancer

All clinical and pathological factors were analysed and compared with serum concentrations of the 35 analytes (Table 5). Advanced T-stage breast cancer (T3 and T4) showed increased serum levels of EGF and ApoH, but decreased levels of A1AT. Patients with lymph node metastasis showed increased serum concentrations of sVCAM-1 and transthyretin. The number of lymph node metastases was positively correlated with sVCAM-1 and D-dimer (Pearson's correlation coefficient = 0.23, *P *= 0.025; and Pearson's correlation coefficient = 0.25, *P *= 0.028, respectively). Patients with distant metastasis showed lower levels of proApoA1 in the serum. The expression status of oestrogen receptor, progesterone receptor and c-erbB2 in tumour tissue was also reflected in serum levels of analytes. In oestrogen receptor-positive patients, serum levels of A2M and HMWK were increased, and concentrations of vitronectin, transthyretin and PAI-1 were decreased. Higher serum levels of A2M and CA19-9 were observed in progesterone receptor-positive patients. In patients showing c-erbB2 expression in tumour tissue, serum levels of sVCAM-1 were decreased, whereas SAA levels were increased. No correlation was noted between the serum EGF level and c-erbB2 expression (c-erbB2 positive = 324 ± 203.58 pg/ml, vs. c-erbB2 negative = 319.39 ± 209.47 pg/ml, *P *= 0.908). The serum concentration of 35 analytes was not influenced by factors such as nuclear grade, histological grade, location or multiplicity of tumour mass.

**Table 5:** Clinicopathological comparison of serum biomarkers in breast cancer

Clinicopathological Concentration of biomarkers factors (mean ± SD)			*P *value
T stage	T1 to 2 (n = 85)	T3 to 4 (n = 13)	
A1AT (ng/ml)	4059.69 ± 6175.49	550.85 ± 712.09	0.001
EGF (ng/ml)	265.71 ± 200.59	452.23 ± 221.82	0.017
ApoH (μg/ml)	122.83 ± 30.97	144.18 ± 31.13	0.023
			
N stage	N0 (n = 32)	N1 to 3 (n = 66)	
sVCAM-1 (ng/ml)	672.13 ± 109.22	755.17 ± 188.23	0.007
Transthyretin (ng/ml)	160.62 ± 55.99	185.54 ± 58.18	0.047
			
M stage	M0 (n = 90)	M1 (n = 8)	
proApoA1 (μg/ml)	27.38 ± 9.28	19.63 ± 13.41	0.032
			
ER expression	Negative (n = 52)	Positive (n = 44)	
A2M (μg/ml)	66.73 ± 23.27	82.51 ± 31.51	0.006
Vitronectin (ng/ml)	5458.08 ± 3224.21	4126.95 ± 1508.43	0.012
Transthyretin (ng/ml)	187.61 ± 57.12	162.24 ± 51.31	0.025
HMWK (ng/ml)	16767.50 ± 12263.40	22483.41 ± 14115.87	0.035
PAI-1 (ng/ml)	20.22 ± 7.06	17.51 ± 5.68	0.043
			
PR expression	Negative (n = 34)	Positive (n = 62)	
A2M (μg/ml)	65.59 ± 19.03	78.56 ± 31.52	0.031
CA19-9 (U/ml)	27.16 ± 19.19	78.89 ± 187.35	0.035
			
c-erbB2 expression	Negative (n = 42)	Positive (n = 53)	
sVCAM-1 (ng/ml)	770.21 ± 197.73	696.91 ± 143.80	0.047
SAA (ng/ml)	2427.74 ± 250.48	5813.77 ± 11884.32	0.048

A1AT = alpha-1 antitrypsin; A2M = alpha-2 macroglobulin; Apo = apolipoprotein; CA = cancer antigen; EGF = epidermal growth factor; ER = oestrogen receptor; HMWK = high-molecular-weight kininogen; PAI = plasminogen activator inhibitor; PR = progesterone receptor; ProApo = proapolipoprotein; SAA = serum amyloid A; SD = standard deviation; sVCAM = soluble vascular cell adhesion molecule.

## Discussion

### Implications and limitations of the present study using an antibody-bead array platform for breast cancer proteomics

Breast cancer is a global issue in that it is the most frequently diagnosed cancer and the leading cause of cancer death among women worldwide [25]. In order to improve survival rates, clinicians need to be armed with new diagnostic modalities capable of detecting breast cancer at an early stage before tumour cells spread to regional lymph nodes or distant sites [26]. Novel cancer biomarkers are expected to open up a new era in cancer diagnostics and will meet current medical needs related to early detection, monitoring and prediction of treatment results in breast cancer patients [27]. To our knowledge, our study is the only one to date showing that the bead array platform is useful for finding signatures specific for primary non-metastatic breast cancer and differentiating these patients from normal subjects using sensitive combinatorial classifiers. This approach also has potential applications for early detection of breast cancer. It is notable that analysis of diverse proteins in serum revealed biomarkers correlating with clinical and pathological variables, including receptor expression status on the cancer cells. Recently, Carlsson and colleagues introduced an scFv-antibody array platform that could successfully distinguish metastatic breast cancer patients from normal people [21]. Nolen and colleagues used a multiplex bead array platform to profile serum biomarkers predicting response to neoadjuvant chemotherapy in locally advanced breast cancer [22]. Thus, in the long term, biomarkers and array-based technology can practically be used for early detection of breast cancer and for stratifying patients, determining their likelihood of experiencing recurrence or having a drug response, or predicting their survival expectancy [16]. Our study has some limitations. Some of the markers identified in this study may not be specific for breast cancer and may possibly reflect a systemic response to tissue damage or inflammation. Furthermore, the analytes included in this study are not comprehensive. A variety of other analytes might behave differentially in the blood of cancer patients. Signature profiling of other benign breast conditions or systemic diseases and further array panel study using a wider range of markers will resolve such issues.

### Alteration of cytokines and growth factors in breast cancer

Among the cytokines and growth factors included in this study, EGF was the only marker increased in the serum of breast cancer patients and correlated with advanced T stage. Up-regulation of other cytokines was not pronounced. High levels of circulating EGF were reported in serum samples from HER2-negative breast cancer patients, although increased levels of IL-8 were consistently noted in serum samples from metastatic breast cancer patients [21,28,29]. This discordant result might be caused by differences in the study populations. In previous studies by Vazquez-martin and colleagues [28] and Carlsson and colleagues [21], serum samples were taken principally from patients with metastasis. However, only 8 of 98 breast cancer patients (8.1%) in this study had metastasis. In the study by Benoy and colleagues relatively large numbers of breast cancer patients without metastasis were recruited and compared with normal control subjects [29]. This difference in study populations might explain the discordant results across the studies with regard to IL-8 and EGF levels.

### Alteration of coagulation and thrombosis in breast cancer

Hypercoagulability is frequently seen in the setting of cancer, with Trousseau's sign first reported over 100 years ago [30]. Multiple mechanisms are considered contributory to this phenomenon, such as secretion of tissue factor, cancer pro-coagulant, PAI-1, mucin molecules with altered glycan and other thrombogenic cytokines from cancer cells [31]. The multiplex array used in this study contained coagulation- and thrombosis-related markers such as sCD40L, HMWK, D-dimer, PAI-1 and free haemoglobin. An assay using this panel revealed increased concentrations of sCD40L and decreased levels of HMWK and PAI-1 in breast cancer patients. Roselli and colleagues first noted the association between elevated plasma sCD40L levels in lung cancer; specifically advanced squamous cell carcinoma. They also noted *in vivo *platelet activation with this type of tumour [32]. Membrane-bound CD40L, a precursor of sCD40L, is a transmembrane glycoprotein mainly expressed by activated T cells and activated platelets [33]. Recently, it has been suggested that activation of the CD40/CD40L pathway may enhance the pro-coagulant activity of tumour cells through up-regulation of tissue factor expression [34]. Thrombin generation and peritumoural fibrin deposition induced by tissue factor then promote angiogenesis and platelet activation [35]. In our study, quantitative changes in serum HMWK levels were also observed in breast cancer patients. This is in agreement with a study previous reporting down-regulation of HMWK in tissue samples from breast cancer patients [36]. Given the fact that HMWK also has pro-thrombotic and pro-angiogenic properties through releasing bradykinin [37], the behaviour of these two proteins in the serum of breast cancer patients is consistent with the perceived concept of cancer biology.

PAI-1 is frequently up-regulated in cancer cells [31], and elevated PAI-1 has been found to be a poor prognostic marker in the setting of breast cancer [38]. PAI-1 contributes to cancer dissemination by preventing excess degradation of the extracellular matrix, modulating cell adhesion [39], promoting tumour angiogenesis [40] and stimulating proliferation [41]. However, in our study, serum PAI-1 levels were unexpectedly decreased in breast cancer patients compared with normal subjects. This may have been due to pre-analytic or analytic error, in addition to other possibilities. PAI-1 exists in plasma or serum as a free form, a complex form mostly with vitronectin and tissue-type plasminogen activator (tPA) or urokinase-type plasminogen activator (uPA), a latent form and a cleaved form [42]. One report described different PAI-1 glycosylation patterns, depending on cellular origin [43]. There are currently no data available concerning changes in amount or concentration of cleaved or variant glycoforms of PAI-1 in cancer patients. The specificity of the antibodies used in our study should be tested, as should qualitative alterations of PAI-1 in cancer that may affect antigenicity of epitopes.

D-dimer is a marker of ongoing fibrinolysis that is frequently increased in various cancers [44-46]. Although the difference in the serum D-dimer concentration between breast cancer and normal patients did not reach statistical significance in our study, the mean D-dimer level was higher in breast cancer patients, and two algorithms (RF and SVM) selected D-dimer as a classifier specific for breast cancer. It is also noteworthy that the serum concentration of D-dimer was correlated with the number of lymph nodes with tumour metastases in our sample.

### Alterations of adhesion molecules in breast cancer

Soluble variants of cell adhesion molecules (CAMs) are elevated in the blood of patients with inflammation, arthritis, diabetes and various cancers [47]. It has been suggested that soluble forms of these CAMs may play an important role in cancer cell growth and metastasis by promoting angiogenesis [48]. As expected based on *in vitro *results, an *in vivo *study on alterations in soluble CAMs in breast cancer showed increased concentrations of sICAM-1 and sVCAM-1 in the blood of advanced breast cancer patients, which was correlated with the number of metastases and the number of circulating tumour cells [49]. However, our study showed somewhat different behaviour on the part of sICAM-1 and sVCAM-1. The concentration of sVCAM-1 was not increased (it was even lower in breast cancer patients), and its level did not covariate with stage or presence of metastasis. There was no meaningful relationship between serum sICAM-1 levels and clinicopathological parameters. However, our study was consistent with previous studies in that the serum level of sVCAM-1 was higher in patients with lymph node metastasis. Before the interpretation of sCAM data, it was thought that sICAM-1 and sVCAM-1 fluctuated widely throughout the menstrual cycle (not the menstruation period) to the degree that the mean difference between the peak and baseline serum levels was up to 20% [50]. It is possible that this type of factor confounded the results in this study. Control of this confounder is necessary in all studies on soluble CAMs so data can be correctly interpreted and the exact behaviour of soluble CAMs can be determined.

Vitronectin is a component of the ECM that is involved in cancer cell adhesion and migration through interaction of its receptor integrin alphavbeta5 or alphavbeta3 [51,52], urokinase-type plasminogen activator receptor (uPAR) complex [53] and PAI-1 [54]. It has recently been shown that matrix metalloproteinase (MMP)-2 secreted by tumour cells degrades vitronectin and produces fragmented vitronectin, which is more potent than its naïve form in promoting adhesion and migration of cancer cells [55]. Fragmented vitronectin is increased in the serum of hepatocellular carcinoma patients, but mRNA expression of vitronectin is paradoxically decreased in carcinoma tissue [56]. In our study, only the naïve form was measured by a pair of capture and detection antibodies used in the 35-plex panel. Given the perceived role of vitronectin in cancer, decreased vitronectin levels in serum might be a reflection of increased turnover rate of vitronectin by tumour cells.

### Alteration of metabolic markers in breast cancer

Proteins related to lipid metabolism are included in the current array panel. Among these markers, decreased expression of ApoA1 was notable in cancer serum. ProApoA1 levels were increased among cancer patients; lower proApoA1 levels were correlated with the presence of metastasis. Down-regulation of ApoA1 is a consistent finding in serum or tissue in the setting of several types of cancer [57,58], and our study validated this phenomenon in the serum of breast cancer patients. ProApoA1 expression was found to be aberrantly increased in tissues from breast cancer patients [59]. Our study also confirmed up-regulation of this protein in serum. ApoA1 is a major lipoprotein component of high-density lipoprotein (HDL) and is also involved in its biogenesis [60]. Recent research on the relationship between blood lipid profiles and breast cancer have shown that HDL-cholesterol level is lower in cancer patients [61], and this decrease is related to up-regulation of mitogens like oestrogen and higher breast cancer risk, especially in overweight and obese women [62]. Thus, ApoA1 like HDL-cholesterol might be a marker reflecting an unfavourable metabolic environment predisposing to breast cancer. The biological and clinical implications of these metabolic markers should be further investigated.

### Alterations of carrier proteins in breast cancer

VDBP, macrophage-activating factor and group component-globulin have diverse biological functions, such as transportation of vitamin D, actin scavenging, induction of chemotaxis with C5a and activation of macrophages [63]. The previous study investigating this protein found that alpha N-acetyl galactosaminidase, which is increased in the blood of cancer patients, is secreted by cancer cells and this enzyme strips the glycosyl moiety of VDBP [64]. The deglycosylated variant loses its macrophage-activating activity, and this occurrence is thought to play an important role in the immune suppression commonly observed in cancer patients. Currently, there are not enough data related to alterations in blood VDBP levels in cancer patients to draw any decisive conclusions, and more information is needed concerning the behaviour of this protein in the setting of cancer.

## Conclusions

This study demonstrated the usefulness of the antibody-bead array approach in finding signatures that may be specific for primary non-metastatic breast cancer and illustrated the potential for early detection of breast cancer. This approach also revealed serum markers related to clinical and pathological features, including receptor expression status in tissue and provided more general systemic information concerning responses in breast cancer patients. Further validation is required before the multiplex bead array approach is routinely used for screening, monitoring, prediction and prognosis purposes.

## Abbreviations

2D-PAGE: two-dimensional polyacrylamide gel electrophoresis; A1AT: alpha-1 antitrypsin; A2M: alpha-2 macroglobulin; AFP: alpha-fetoprotein; Apo: apolipoprotein; AUC: area under the curve; BSA: bovine serum albumin; CA: cancer antigen; CAM: cell adhesion molecules; CEA: carcinoembryonic antigen; CRP: C-reactive protein; EGF: epidermal growth factor; ELISA: enzyme-linked immunosorbent assay; HDL: high-density lipoprotein; HER: human epidermal growth factor receptor; HMWK: high-molecular-weight kininogen; HSP: heat shock protein; IL: interleukin; LDA: linear discriminant analysis; MALDI: matrix-assisted laser desorption/ionisation; MPO: myeloperoxidase; MS: mass spectrometry; PAI-1: plasminogen activator inhibitor-1; PBS: phosphate-buffered saline; PCA: principal component analysis; ProApo: proapolipoprotein; PSA: prostate specific antigen; RF: random forests; ROC: receiver operating curve; SAA: serum amyloid A; sCD40L: soluble CD-40 ligand; SELDI-TOF: surface-enhanced laser desorption/ionisation time-of-flight; sICAM-1: soluble intercellular adhesion molecule-1; sVCAM-1: soluble vascular cell adhesion molecule-1; SVM: support vector machine; VDBP: vitamin-D binding protein.

## Competing interests

PP, YS, SO, DN and CK are stock holders of BioInfra Inc., Seoul, Korea. PP, YS, WL, KL, SY, HH and KK are employees of BioInfra Inc. and have received salary from it. BioInfra Inc. is currently applying for a patent relating to the biomarkers found and described in this manuscript.

## Authors' contributions

BK and JWL contributed equally to conception, design and interpretation of the study and drafting of the manuscript. PP and YS contributed to conception and design. WL, KL, SY and HH contributed to construction of the multiplex panel and acquisition of data. KK, YK and SO contributed to statistical analysis.
